# Sporulation in soil as an overwinter survival strategy in *Saccharomyces cerevisiae*

**DOI:** 10.1093/femsyr/fov102

**Published:** 2015-11-13

**Authors:** Sarah J. Knight, Matthew R. Goddard

**Affiliations:** 1 School of Biological Sciences, The University of Auckland, Auckland 1142, New Zealand;; 2 School of Life Sciences, University of Lincoln, Lincoln, LN6 7DL, UK

**Keywords:** fruit forest-reservoir hypothesis, microbial ecology, *Saccharomyces cerevisiae*, sporulation, yeast, ecology

## Abstract

Due to its commercial value and status as a research model there is an extensive body of knowledge concerning *Saccharomyces cerevisiae*'s cell biology and genetics. Investigations into *S. cerevisiae*'s ecology are comparatively lacking, and are mostly focused on the behaviour of this species in high sugar, fruit-based environments; however, fruit is ephemeral, and presumably, *S. cerevisiae* has evolved a strategy to survive when this niche is not available. Among other places, *S. cerevisiae* has been isolated from soil which, in contrast to fruit, is a permanent habitat. We hypothesize that *S. cerevisiae* employs a life history strategy targeted at self-preservation rather than growth outside of the fruit niche, and resides in forest niches, such as soil, in a dormant and resistant sporulated state, returning to fruit via vectors such as insects. One crucial aspect of this hypothesis is that *S. cerevisiae* must be able to sporulate in the ‘forest’ environment. Here, we provide the first evidence for a natural environment (soil) where *S. cerevisiae* sporulates. While there are further aspects of this hypothesis that require experimental verification, this is the first step towards an inclusive understanding of the more cryptic aspects of *S. cerevisiae*'s ecology.

## Abstract (precis)

The ability of *Saccharomyces cerevisiae* to sporulate in soil supports a ‘fruit forest-reservoir’ life-cycle.

## INTRODUCTION

*Saccharomyces cerevisiae* is arguably one of the world's most important microbes due to its use in beer, wine and bread production, various biotechnological applications, and its premier research model status (Chambers and Pretorius 2010; Dujon 2010; Gray and Goddard 2012; Hittinger 2013; Hyma and Fay 2013). Despite the vast amount of information concerning *S. cerevisiae*'s molecular biology, comparatively little is known about its ecology, which is not only a worthy pursuit in its own right, but also imperative to help put the swathes of genetic and molecular information gained from this species into context. While the genetic and laboratory conditions under which *S. cerevisiae* sporulates are extremely well described, we are unaware of any report describing the environments that might promote sporulation in nature (Neiman 2011). Here, we provide the first report of this and show that soil promotes sporulation in *S. cerevisiae*.

To begin to understand the ecology of this budding yeast, it is important to appreciate its life-cycle, which has been exclusively determined by observation in the laboratory. In nutrient rich environments diploid cells replicate vegetatively via budding. Populations of yeasts may be propagated mitotically for thousands of generations, at least in the lab where nutrients are plentiful (Buckling *et al*. 2009). When nitrogen and fermentable carbon sources such as glucose are absent, and a non-fermentable carbon source such as acetate is present, diploid cells containing both *MAT*a and *MAT*α mating types undergo sporulation: this comprises a meiotic division, with recombination, to produce four haploid spores, two of each mating type, encased in an ascus, which is known as a tetrad (Esposito and Klapholz 1981; Honigberg and Purnapatre 2003; Neiman 2005, 2011; Piccirillo and Honigberg 2010). When spores encounter sufficient nutrients, they germinate and diploid cells are formed by the fusion of two haploid cells of opposite mating type. If a haploid germinated spore fails to encounter another haploid of the opposite mating type, then after a couple of divisions the mother cell may switch mating type (homothallism), and mate with a daughter cell to produce an entirely homozygous diploid. If this mate type switching system is non-functional (heterothallism), haploid cells divide mitotically until a spore of the opposite mating type is encountered.

### How *S. cerevisiae*'s life cycle fits its ecology

It is not yet clear how *S. cerevisiae*'s laboratory inferred life cycle fits with its ecology in natural environments. The fermentation of fruits, principally those gathered by humans, is currently the only habitat from which *S. cerevisiae* has been isolated without the need for enrichment (Goddard and Greig 2015). *Saccharomyces cerevisiae* is well documented to actively grow and infest fruit juice, and is capable of dominating the microbial community once fruit is gathered and crushed via more rapid growth and the ecosystem engineering effects of fermentation (Pfeiffer, Schuster and Bonhoeffer 2001; Merico *et al*. 2007; Goddard 2008; Goddard and Greig 2015). However, *S. cerevisiae* is very rare on fruits prior to them being gathered and crushed by humans, and metagenomic analyses of fruit epiphytes show *Saccharomyces* is just ∼1:20 000 of the fungal community (Taylor *et al*. 2014). Since fruits are present for only a fraction of the year, presumably a mechanism has evolved to ensure *S. cerevisiae's* survival when sugar rich fruit is not available. However, the locations of other habitats, what form *S. cerevisiae* takes within them and how it survives generally until the next season of fruit, are not clear (Goddard and Greig 2015).

A number of studies have isolated *S. cerevisiae* from a variety of habitats, but other than active ferments, the only habitats from which this species has been consistently isolated appears to be oak bark and soil (Goddard and Greig 2015). However, soil and tree bark may not represent a niche to which *S. cerevisiae* is adapted, but might simply reflect yeast ecologists sampling preferences (Goddard and Greig 2015). A recent report shows that *S. cerevisiae* is present at reasonable abundance and can survive in the nests of overwintering social wasps (Stefanini *et al*. 2012). In addition, *S. cerevisiae* is associated with *Drosophila* and other insects (Goddard *et al*. 2010; Buser *et al*. 2014). However, isolation from all niches other than fruit juice that has been artificially concentrated by humans requires enrichment as *S. cerevisiae* is in such low abundances generally in the environment (Mortimer and Polsinelli 1999; Serjeant *et al*. 2008). This has led to a neutral nomad hypothesis for *S. cerevisiae*: that it is not necessarily a fruit specialist, but a generalist that exists at low frequencies in many niches (Goddard and Greig 2015).

Whether *S. cerevisiae* exists as spores or vegetative cells in habitats other than ferments is masked by the enrichment procedure that is necessary to isolate it, as this causes both the growth of vegetative cells and germination and growth of spores in original samples. As far as we are aware no environment outside the laboratory has been assayed for its ability to induce sporulation. Among the niches from which *S. cerevisiae* has been isolated, the conditions where sporulation is more likely to be induced are those where nutrients are comparatively low. Thus, one obvious hypothesis is that cells transition into a sporulated state when the fruit season ends and nutrients are depleted. Selection is predicted to have operated on an increased propensity to sporulate under these conditions as it provides cells with increased protection against harsh and relatively poor nutrient conditions experienced over winter. Since *S. cerevisiae* does not demonstrate any growth in a sporulated state, selection is coarse in that it will only act to determine whether spores survive or not and will be impotent in any more subtle manipulations of the genetic variance in this species.

While the genetic determinants of sporulation have been extremely well characterized, the function of sporulation is still not clear. Stationary phase diploids cells are reasonably tough, and while spores are more resistant to a range of chemical and physical insults in the laboratory (such as ether and heat), it is not clear how or even if these reflect natural conditions (Neiman 2011). Spores are no more resistant to more ‘natural environment’ like conditions such as freeze–thaw and desiccation than stationary phase cells (Coluccio *et al*. 2008). One significant observation is that spores are more resistant to mild acid and alkali conditions and to digestive enzymes, and this fits nicely with the observation that spores are better at surviving passage through *Drosophila melanogaster* digestive tracks (Reuter, Bell and Greig 2007; Coluccio *et al*. 2008). Recent work has substantiated old observations that *S. cerevisiae* is not only associated with but actively attracts *Drosophila* with volatile metabolites (Buser *et al*. 2014; Christiaens *et al*. 2014; Palanca *et al*. 2013); however, we are aware of no evidence that passage through insect guts promotes sporulation—indeed vegetative cells mostly die (Reuter, Bell and Greig 2007). Thus, presumably cells must have sporulated prior to consumption if they are to survive. While this provides potential evidence for a function of spore formation (to survive insect ingestion), it does not necessarily mean that is the function for which sporulation was selected and thus primarily adapted. Sporulation efficiencies among strains are known to vary greatly, and few if any are able to achieve 100%. There are very few inferences of *S. cerevisiae*'s frequency of meiosis in the natural environment (Ruderfer *et al*. 2006; Magwene *et al*. 2011), and no direct estimates that we are aware of, but the consensus is that it is ‘rare’ but still plays an important role in the genetic structure and evolution of the species. Experimental evolution shows some *S. cerevisiae* decline in their ability to sporulate when propagated mitotically (Zeyl *et al*. 2005). That, to date, most cells found in nature have been diploid and capable of sporulating suggests that selection has been strong enough to maintain this trait, but again the ecological conditions that promote sporulation are elusive. Overall, these observations do not explain why sporulation need be associated with meiosis (sex). Experiments that have used *S. cerevisiae* to test the fundamental question of why sex is maintained support Weismann's original idea that sex's advantage lay in the fact that it increases genetic variance, and thus rates of adaptation (Burt 2000; Goddard, Godfray and Burt 2005). Directional selection (adaptation) is likely stronger in novel environments, and this links with dispersal as yeasts have no control over the habitats they are dispersed too, and from this perspective it makes sense that sporulation is linked with dispersal.

### The fruit forest-reservoir hypothesis

**Figure 1.**
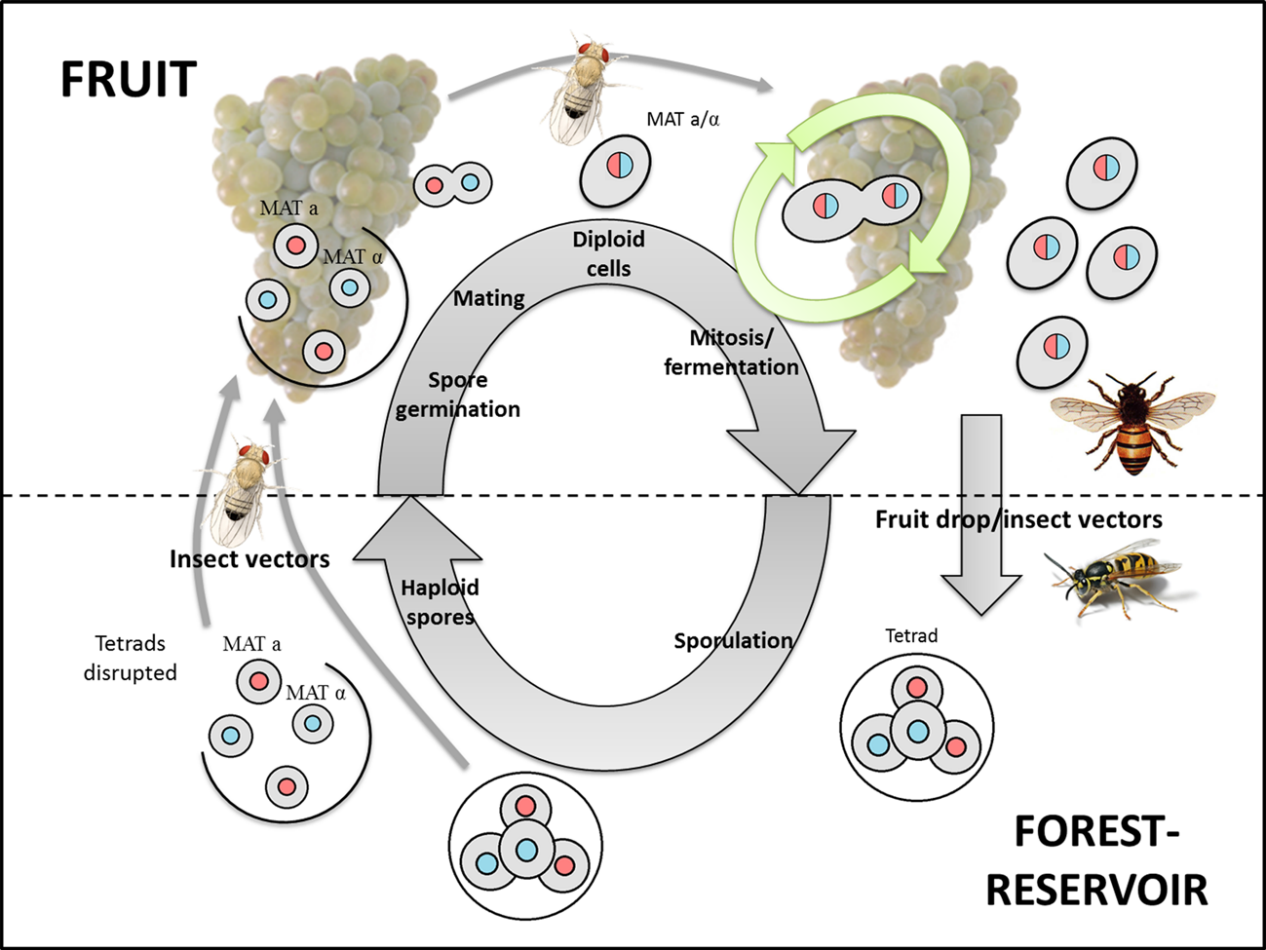
The fruit forest-reservoir hypothesis. A reservoir population of *S. cerevisiae* exists in various forest habitats (soil, bark, etc.) primarily in a sporulated state, and that some fraction of these spores are transported to fruit, potentially by insect vectors. These spores germinate and mate in the sugar rich fruit niche to form diploid cells that undergo mitosis and fermentation. At the end of the fruit season some fraction of the now very large population are returned and contribute to the forest reservoir, and the cycle continues.

By combining the experimental data and observations outlined above, and building on the ideas presented by Goddard *et al*. (2010), we introduce the ‘fruit forest-reservoir hypothesis’ (Fig. 1). The proposed cycle begins with the concept that *S. cerevisiae* exists as a diffuse low abundance reservoir in various forest niches such as soil and tree bark in a sporulated state. There is good evidence showing that *S. cerevisiae* is present in forest niches, including insect nests, at low frequencies (Sniegowski, Dombrowski and Fingerman 2002; Sampaio and Gonçalves 2008; Goddard *et al*. 2010; Zhang *et al*. 2010; Hyma and Fay 2013; Knight and Goddard 2015). Isolates from non-fruit niches typically tend to be homozygous, where those from fruit ferments tend to be more heterozygous (Diezmann and Dietrich 2009; Goddard *et al*. 2010; Magwene *et al*. 2011; Knight and Goddard 2015). This observation is in line with the idea that enrichment procedures may have caused rare spores to germinate and achieve a homozygous diploid state after mate-type switching (Goddard *et al*. 2010). Such observations provide only weak correlational support for this idea though. Experimental evidence that *S. cerevisiae* exists as spores in forest-associated niches does not exist. We hypothesise that some fraction of this low abundance but diffuse forest-reservoir is transferred to fruits when they come into season, potentially by insects (Mortimer and Polsinelli 1999; Reuter, Bell and Greig 2007; Stefanini *et al*. 2012; Palanca *et al*. 2013; Buser *et al*. 2014; Christiaens *et al*. 2014;). Some of these initially rare insect-vectored *S. cerevisiae* are deposited on/in fruit, infect them once ripe and damaged, and eventually come to dominate and achieve large populations. While many studies have shown *S. cerevisiae* may invade homogenized fruit juices gathered by humans and transported to wineries, and come to dominate from initially being rare (Mortimer and Polsinelli 1999; Xufre *et al*. 2006; Goddard 2008), evidence that the same occurs in and on fruit in natural ecosystems is lacking. Recent work shows that some volatiles produced by growing *S. cerevisiae* attract *Drosophila*, and this is one vehicle by which *S. cerevisiae* might escape from ephemeral fruits (Palanca *et al*. 2013; Buser *et al*. 2014; Christiaens *et al*. 2014;). Finally, at the end of the fruiting season, some fraction of the population are returned and contribute to the forest-reservoir population, potentially with the fruit as it drops, where they sporulate and await the next, or some subsequent season of fruit for the cycle to commence turning.

While some aspects of the ‘fruit forest-reservoir hypothesis’ appear supported by previous experimental observations, there are many components that are elusive and require proper evaluation. One crucial aspect relies on determining the environments in which *S. cerevisiae* sporulates. We test how the presence of soil nutrients affects sporulation efficiency in twelve genetically diverse genotypes of *S. cerevisiae* isolated from both vineyard soil and the ferment of fruits (Knight and Goddard 2015) with the aim of taking steps forward in our understanding of the more cryptic aspects of *S. cerevisiae*'s ecology.

## METHODS

### Genotype selection and preparing cultures

Six genotypes isolated from vineyard soil and six isolated from spontaneous *Vitis vinifera* var. Sauvignon Blanc ferments were selected for analysis from those described in (Knight and Goddard 2015). These genotypes were selected on the basis of maximal genetic differentiation as ascertained by microsatellite genotyping at eight loci (Knight and Goddard 2015). None of the genotypes are genetically similar to a diverse set of international isolates (Liti *et al*. 2009) or to commonly used commercial strains and are therefore considered to be derived from the New Zealand population. However, from analyses using previously isolated NZ strains from vineyard soil, bark and flowers, the New Zealand population appears reasonably closely related to the wine/European group (Cromie *et al*. 2013). All isolates were stored at −80°C in 15% glycerol and were revived in 10 mL liquid YPD (1% yeast extract, 2% peptone, 2% glucose) at 25°C. Once each culture reached an optical density of 0.6 at a wavelength of 600 nm (about the point where the cells are mid exponential phase) it was centrifuged at 3000 rpm for three minutes and washed twice with 10 mL of sterile water, centrifuging to pellet the cells between each wash. The cells were resuspended in 1 mL of sterile water, ready for plating.

### Soil agar

We attempted to observe cells directly in soil with standard microscopy, but were unable to differentiate deliberately inoculated cells from soil particles, other debris, and other microbes naturally present. Thus, we developed a soil agar media designed to emulate the natural conditions in soil while still permitting the observation of cells. The composition and analytical parameters of the soil used are provided in Table S1 (Supporting Information). 50–200 g of dry soil from Mate's Vineyard at Kumeu Wine Estate (West Auckland, New Zealand) was placed in 1 L of distilled water, rocked at room temperature for six hours and settled overnight at 4°C. The supernatant was poured off to separate it from the larger soil particles and then filtered with a 40 μm cell strainer. Dimethyl dicarbonate (DMDC) was used to sterilise the ‘soil tea’ in two doses: first at a concentration of 200 μL L^−1^ with stirring for six hours, then at 400 μL L^−1^ with stirring overnight. The sterilized soil ‘tea’ was subsequently mixed with an autoclaved agar solution to create soil agar plates with a final agar concentration of 1.5%.

### Initial sporulation study

100 μL of exponential-phase cell solutions of each genotype were plated in triplicate on synthetic grape juice media (SGM) agar (recipe is based on Harsch *et al.* (2009) and is provided in Supplementary Table S2 with the addition of 1.5% agar to solidify), sporulation agar (1% potassium acetate, 0.1% yeast extract and 0.05% glucose, 1.5% agar), plain agar (1.5% agar) and soil agar (final concentration of 25 gL^−1^ soil tea and 1.5% agar) and incubated at 25°C. After 2 days and 2 weeks, the proportion of sporulated cells in each population was calculated by scraping the surface of the agar with a sterile tooth pick, resuspending in sterile water, visualizing with a light microscope and scoring at least 100 cells for each sample. Each cell was scored as either sporulated or not sporulated. Ambiguously sporulated cells were not included in the count.

### Time course study

100 μL of exponential-phase cell solutions of each genotype were plated in triplicate on plain and soil agar (final concentration of 100 gL^−1^ soil tea and 1.5% agar) and incubated at 25°C. Due to observations of two-spored asci in the first experiment, the number of unsporulated cells, four-spored asci (tetrads) and two-spored asci (dyads) were counted each day for eight days by scoring over 150 cells from each plate (as above, ambiguously sporulated cells were not counted).

### Statistical analyses

As proportion data have heterogeneous variance, all data underwent arcsine transformation prior to analyses (Sokal and Rohlf 1995). A linear mixed effects model with niche of isolation and sporulation environment as fixed effects and genotype as a random effect was employed to evaluate individual time points using JMP (version 11). Non-linear asymptotic exponential two and three parameter growth models, and generalized linear models with logit transformation, were employed to evaluate sporulation dynamics. The three-parameter model used was *y* = *a* − *b* e^−^^cx^, where *x* and *y* are time and proportion sporulated, and *a, b* and *c* the three parameters. Model fitting and comparisons were conducted in R (version 3.3.2) using least squares and maximum likelihood methods, and the ‘anova()’ command for model comparisons which implement a chi-squared test, following Crawley (2013).

## RESULTS

**Figure 2.**
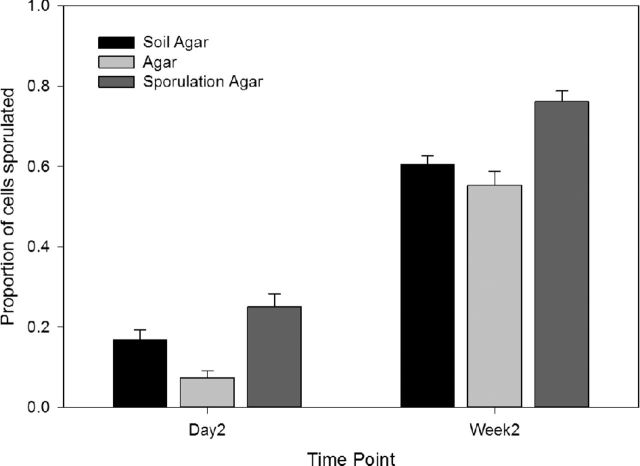
The mean proportion of sporulated cells in each environment. The data for synthetic grape juice is not shown as no sporulation was observed for any *S. cerevisiae* genotype. The error bars represent the standard error around each mean. Sporulation proportions significantly differ across all environments at both time points (F_2,92_ = 39.8 and 28.5; both *P* < 0.0001).

The first experiment evaluated if there was any effect of soil extract on the propensity of *S. cerevisiae* cells to undergo sporulation. Three controls were used in this analysis including standard laboratory sporulation media as a positive control, plain agar to account for any effect that agar alone might have, and a SGM as a proxy for a nutrient rich fruit environment. A total of 288 sporulation estimates were gathered across two time points for 12 genotypes in four environments and these are available in Supplementary Dataset 1. No sporulation was observed by any strain at either time point for any the 7300 cells scored in the SGM environment. Statistical analyses revealed a significant effect of environment on sporulation in the remaining three environments at days 2 and 14 (F_2,92_ = 39.8 and 28.5, respectively; both *P* < 0.0001). The niche from which strains were originally isolated had no significant effect on sporulation at either time point (F_1,10_ = 0.81 and 0.60; *P* = 0.39 and 0.46), nor was there a significant interaction between sporulation environment and the original niche of isolation (F_3,92_ = 0.52 and 97; *P* = 0.59 and 0.38). The average proportion of cells sporulated for each time point and environment (except SGM as no sporulation was observed for any genotype on this media) can be seen in Fig. 2, and histograms showing variance in sporulation by both sporulation environment and strain origin are shown in Figs S1 and S2 (Supporting Information). Subsequent Tukey HSD (α = 0.05) analysis shows that all environments are significantly different from each other in terms of the extent of sporulation they elicit, with the standard laboratory sporulation media inducing the greatest sporulation, followed by soil agar, plain agar, and lastly, the SGM which did not induce sporulation at all.

**Figure 3.**
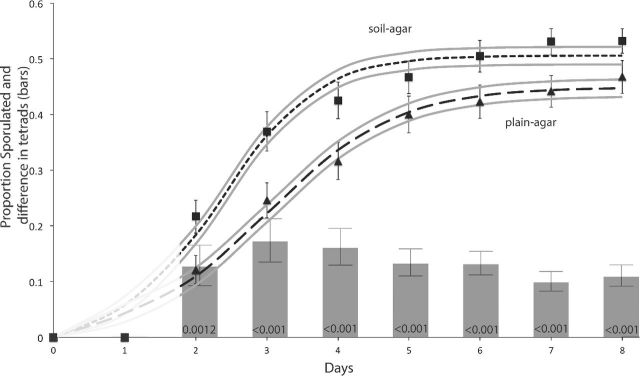
Sporulation dynamics across eight days, showing the mean (± s.e.) in plain-agar (triangles) and soil-agar (squares) environments. The best-fit three-parameter asymptotic exponential model is shown for both: soil-agar = small dash; and plain-agar = long dash. The standard error for each curve is shown as light grey lines. The model is greyed out prior to 2 days as no sporulation was recorded. The histogram is at the same scale as the rest of the plot and shows the mean (± s.e.) difference in the proportion of tetrads between the two environments at each time point: the positive values show greater tetrad number of in the soil-agar environment. The probability values for *t*-tests evaluating whether the difference in tetrad proportions are different from zero (i.e. that tetrad proportions do not differ between plain- and soil-agar environments) are shown at the bottom of each bar and have been adjusted for multiple comparisons using the Benjamini–Hochberg method (Benjamini and Hochberg 1995).

The second time course study tested sporulation dynamics over eight days for all genotypes in just soil agar and plain agar environments, and comprised 576 sporulation estimates (Supplementary Dataset 2). All genotypes in both sporulation environments exhibited reasonable degrees of sporulation after eight days of incubation. Analyses of the final proportions with a mixed effects linear model show significant differences in the extent of sporulation between soil and plain agar environments (F_1,59_ = 26.116, *P* < 0.0001; Fig. 3; Fig. S3, Supporting Information; Supplementary Dataset 2). However, the more comprehensive analyses evaluates sporulation dynamics—analyses across time, and we chose to use non-linear asymptotic exponential growth models as these encapsulate population change processes that provide biological insight into the rate and extent of sporulation. We determined a three-parameter model was a significantly better fit than a two-paraemeter one (*P* = 1.3 × 10^−9^) to the data overall. The three parameters estimate the ‘lag’ until start of sporulation, the rate of sporulation and the final extent of sporulation (the asymptote). While a three-parameter model adequately describes sporulation dynamics in both environments, the values of all three parameters significantly differ between models fit to each environment individually (*P* < 0.0001). This analysis reveals that sporulation on soil agar has a shorter lag and a greater rate and final extent of sporulation. The fitted models and their standard errors are shown in Fig. 3 along with the mean proportion of sporulation in each environment. In addition, as an alternative approach, we conducted logistic regression on the proportion data using a generalized linear model employing logit transformation with binomial errors drawn from the quasibinomial distribution, as is appropriate for proportions (Crawley 2013). This analysis also reports a significant effect of environment on sporulation dynamics (*P* = 0.008 58). Together these analyses show that soil induces more rapid sporulation and that a greater proportion of cells are sporulated by day eight compared to plain agar.

We noted the presence of dyads as well as tetrads in the first experiment and so differentiated between these in this second time-course experiment. The dynamics are more complicated—the proportion of tetrads peaks early and then drops because the formation of tetrads from the unsporulated fraction of the population is relatively faster than the formation of dyads. The slower accrual of dyads means the relative proportion of tetrads decreases with time and the number of dyads increases. Non-linear analyses make little biological sense, as the question of interest here is the relative difference in dyad versus tetrad formation in the two environments. This difference is shown as bars in Fig. 3, and it can be seen that populations on soil agar contain significantly more tetrads than on plain agar at all-time points after day 2 (all *P* < 0.0012).

## DISCUSSION

Here, we provide evidence that when *S. cerevisiae* is put in a soil environment the rate and extent of sporulation is promoted. The observation that sporulation is greater on soil agar compared to plain agar shows that it is not solely the lack of nutrients that are responsible for sporulation, but that some component of the soil tea itself increases the propensity for sporulation. Over half the cells assayed here sporulated after two weeks on soil agar and this provides experimental evidence to suggest that a reasonable fraction of *S. cerevisiae* residing in the soil do so in a sporulated state. It will be interesting to see how these observations translate to soil with differing characteristics (crucially, different concentrations of organic matter). These data are in line with the hypothesis that a sporulation response promotes self-preservation when in soil; however, this does not show that *S. cerevisiae* is adapted (in the correct sense) to sporulate in soil. Sustained selection for sporulation may have occurred in some other environment, and sporulation in soil may occur as a side effect of this.

From laboratory observations, typically meiosis results in the formation four haploid spores encased in an ascus (a tetrad). However, meiosis may also result in the formation of only two spores—these are known as dyads. Mutations in a number of genes involved in meiosis are known to cause modifications to the spindle pole bodies or outer plaque formation and result in dyad formation (Reviewed in: Neiman 2005). Some mutations that affect spore formation can be dose dependent; for example, cells with two mutant alleles of *MPC70* only produce dyads, while heterozygous cells produce a mix of tetrads and dyads, and cells containing two functional alleles produce primarily tetrads (Wesp, Prinz and Fink 2001). However, stressful environments are also known to affect spore formation with dyads being formed as a metabolic response to a depletion of carbon during meiosis (Davidow, Goetsch and Byers 1980; Neiman 2005; Taxis *et al*. 2005). Rather than arresting meiosis due to a lack of nutrients, depletion of the carbon source (such as acetate) after commitment to sporulation triggers the cell to conserve the remaining available external energy and a switch from forming tetrads to less energy expensive dyads (Davidow, Goetsch and Byers 1980; Neiman 2005; Taxis *et al*. 2005). These dyads are called non-sister dyads (NSDs) as they contain genetic information from homologous chromosomes rather than sister chromatids due to the meiosis II outer plaques only being formed by two of the four spindle pole bodies, one from each spindle (Davidow, Goetsch and Byers 1980; Neiman 2005; Taxis *et al*. 2005; Neiman 2011). The formation of NSDs not only maintains genetic diversity, but ensures two spores of opposite mating type are made, leaving the possibility for sister spores to mate with one other upon germination. We observed both tetrad and dyad formation in all genotypes, and this suggests dyad formation here is not primarily genetically determined. The formation of tetrads occurs earlier in the time course and plateaus, while dyad formation continues to increase (Fig. 3): the reduction in rate of tetrad formation is in line with the switch to greater dyad formation being driven by decreasing nutrients. Thus, we speculate that dyad formation here is a metabolic response, and it is the greater nutrients offered by soil that allow more cells to become tetrads on soil compared to plain agar.

The observation that sporulation occurs on plain agar goes against the evidence that a non-fermentable carbon source is required for sporulation and suggests that either the agar itself contains the required nutrients to initiate sporulation, or that the genotypes tested here regulate sporulation in a manner different to that of closely studied lab strains. Agar is a polysaccharide complex often extracted from red algae, and while it's composition is complex, it has been shown to contain galactose (Duckworth and Yaphe 1971). *Saccharomyces cerevisiae* can ferment galactose, so perhaps the presence of a non-fermentable carbon source is not always necessary for sporulation.

The observation of sporulation in soil is in line with a life history strategy favouring self-preservation and dormancy in unfavourable environments. This observation also provides experimental evidence for soil as one forest habitat harbouring a sporulated reservoir of this species. Experiments have also shown that wasps’ nests are another overwintering habitat for *S. cerevisiae*, but whether cells existed as spores was not determined (Stefanini *et al*. 2012). *Saccharomyces cerevisiae* is also well documented to be associated with fruit flies (e.g. Palanca *et al*. 2013; Buser *et al*. 2014; Christiaens *et al*. 2014). Temperate species of fruit flies typically overwinter as diapausing pupae, entering the soil after leaving the fruit as winter approaches, emerging as adults the following summer (Bateman 1972). Therefore, if *S. cerevisiae* changes into a sporulated state when deposited in soil as the fruiting season ends, flies and wasps may potentially ingest *S. cerevisiae* as spores: these spores are more likely than vegetative cells to survive passage through the insect guts. Passage through flies has been shown to promote outcrossed matings, and thus, insects may not only facilitate dispersal but also increased genetic variance (Reuter *et al*. 2007).

If *S. cerevisiae* cycles between the fruit and soil/other forest niches, then contemporaneous populations occupying these niches should be connected. Population genetic studies investigating *S. cerevisiae* report no evidence for population differentiation between fruit associated and forest niches on small geographic scales in both the northern and southern hemisphere (Goddard *et al*. 2010; Hyma and Fay 2013; Knight and Goddard 2015). Here, we also provide data to support connectivity between these contemporaneous populations by showing no difference in the phenotypic trait of sporulation efficiency between populations originally isolated from soil and the ferments of fruits from the same area at the same time. In contrast, previous findings suggested that genotypes isolated from oak trees were more efficient at sporulating and forming asci with predominantly four-spores compared to genotypes isolated from wine fermentations that formed large numbers of two- and three-spored asci (Gerke, Chen and Cohen 2006). In addition, studies evaluating these same isolates, suggest populations from oak trees and vineyards are genetically different (e.g. Liti *et al*. 2009; Cromie *et al*. 2013). However, these genotypes were isolated from distant locations and different times, with the oak isolates originating solely from North America and the vineyard isolates mostly from wider Europe but also Australia, South Africa and California. Thus, these findings may be equally explained by the fact that they are drawn from populations with markedly different geographic origins, and they are genetically and thus phenotypically different (including in their sporulation ecology) because of a lack of gene flow at large scales. In short, either differential selection and/or genetic drift may cause different subpopulations to diverge. The key to test this would be to isolate the corresponding contemporaneous oak/wild and vineyard/ferment isolates from each of these areas and test them. If the contemporaneous wild and wine populations in different discrete areas are genetically homogenous then this would tend to support the fruit forest-reservoir hypothesis, if they are not then it would tend to reject it. However, it is clear that populations inhabiting different niches in New Zealand are connected, but it remains to be seen if other *S. cerevisiae* populations conform to a fruit forest-reservoir life cycle.

This is one piece of the puzzle investigating the ecology of *S. cerevisiae*, and begins to address the more cryptic phase of its life-cycle. The fruit forest-reservoir is a straw-man hypothesis, and its function is to help us understand better the ecology of this species. It has recently been suggested that *S. cerevisiae* may not be adapted to any niche, but is a nomad that has evolved the ability to survive in many habitats (Goddard and Greig 2015): perhaps it does so by existing as spores in most of them.

## Supplementary Material

Supplementary Data
