# Supplementary material for: Sporulation in soil as an overwinter survival strategy in Saccharomyces cerevisiae
Source: FEMS Yeast Res. 2015 Nov 13;16(1):fov102. doi: 10.1093/femsyr/fov102 (PMC5815064; doi:10.1093/femsyr/fov102)
Supplement: Supplementary Data [file fov102_supplementary_data.zip › Supplementary Files.docx]

**Supplementary Files: Knight and Goddard**

*Two supplementary figures are provided as detailed below:*

**Supplementary Figure 1:** Histograms of the proportion of cells sporulated by media type (agar = plain agar, soil = soil agar and SM = sporulation media) and by time point (day 2 and week 2) for the sporulation study.

**Supplementary Figure 2:** Histograms of the proportion of cells sporulated as in Supplementary Figure 2 and also split by the strains origin of original isolation.

**Supplementary Figure 3:** The average proportion of the total number of cells sporulated as well as the proportion of tetrads and dyads within that total across the eight days of the time course experiment for both the (a) plain agar and (b) soil agar plates. The error bars represent the standard error around each average.

*Two supplementary tables are provided as detailed below:*

**Supplementary Table 1:** The composition and basic analytical parameters of the soil used to make the soil agar. All tests were performed by Eurofins NZ Laboratory Services Limited.

**Supplementary Table 2:** The recipe used here for synthetic grape juice media (SGM), adapted from Harsch *et al.* (2009).

*Two supplementary datasets are provided as detailed below:*

**Supplementary Dataset 1:** The raw counts, proportions of cells sporulated and transformed proportions for each sample in the sporulation study. Note SM refers to sporulation media and SGM refers to synthetic grape juice media.

**Supplementary Dataset S2:** The raw data, proportions and transformed proportions for each sample in the time course study.
