# Supplementary material for: Sporulation in soil as an overwinter survival strategy in Saccharomyces cerevisiae
Source: FEMS Yeast Res. 2015 Nov 13;16(1):fov102. doi: 10.1093/femsyr/fov102 (PMC5815064; doi:10.1093/femsyr/fov102)
Supplement: Supplementary Data [file fov102_supplementary_data.zip › Supplementary Table 1.docx]

**Supplementary Table 1:** The composition and basic analytical parameters of the soil used to make the soil agar. All tests were performed by Eurofins NZ Laboratory Services Limited.

| **Analytical Parameter** | **Measurement** | **Composition** | **Measurement** |
| --- | --- | --- | --- |
| pH | 5.7 | Total Carbon | 2.9 % |
| Calcium, Ca | 10 MAF QT | Organic Matter | 5.1 % |
| Olsen Phosphate, P | 30 µg/mL | Coarse Sand (0.6-2.0 mm) | <1 % |
| Potassium, K | 9 MAF QT | Medium Sand (0.2-0.6 mm) | 8 % |
| Sulfate sulfur, S(SO_4_) | 13 ppm | Fine Sand (0.06-0.2 mm) | 35 % |
| Magnesium (Mg) | 36 MAF QT | Silt (0.002-0.06 mm) | 34 % |
| Sodium (Na) | 9 MAF QT | Clay (<0.002 mm) | 22 % |
