# Supplementary material for: Sporulation in soil as an overwinter survival strategy in Saccharomyces cerevisiae
Source: FEMS Yeast Res. 2015 Nov 13;16(1):fov102. doi: 10.1093/femsyr/fov102 (PMC5815064; doi:10.1093/femsyr/fov102)
Supplement: Supplementary Data [file fov102_supplementary_data.zip › Supplementary Table 2.docx]

**Supplementary Table 2:** The recipe used here for synthetic grape juice media (SGM), adapted from Harsch *et al.* (2009).

| Glucose | 105 gL^-1^ | L-alanine | 100 mgL^-1^ |
| --- | --- | --- | --- |
| Fructose | 105 gL^-1^ | L-arginine-HCl | 484 mgL^-1^ |
| Potassium tartrate hemihydrate (CAS 6100-19-2) | 5 gL^-1^ | L-aspartic acid | 50 mgL^-1^ |
| Malic Acid | 3 gL^-1^ | L-asparagine | 10 mgL^-1^ |
| Citric Acid | 0.2 gL^-1^ | L-cysteine | 5 mgL^-1^ |
| Potassium hydrogen phosphate (K_2_HPO_4_) | 1.14 gL^-1^ | L-glutamic acid | 100 mgL^-1^ |
| Magnesium sulfate (MgSO_4_.7H_2_O) | 1.23 gL^-1^ | L-glutamine | 125 mgL^-1^ |
| Calcium chloride (CaCl_2_.2H_2_O) | 0.44 gL^-1^ | L-glycine | 5 mgL^-1^ |
| Manganese (II) chloride (MnCl_2_.4H_2_O) | 198.2 µgL^-1^ | L-histidine | 20 mgL^-1^ |
| Zinc sulfate (ZnSO_4_.7H_2_O) | 287.5 µgL^-1^ | L-isoleucine | 25 mgL^-1^ |
| Iron (II) sulfate (FeSO_4_.7H_2_O) | 70.1 µgL^-1^ | L-leucine | 25 mgL^-1^ |
| Copper (II) sulfate (CuSO_4_) | 25.3 µgL^-1^ | L-lysine-HCl | 6 mgL^-1^ |
| Boric acid (H_3_BO_3_) | 5.7 µgL^-1^ | L-methionine | 10 mgL^-1^ |
| Cobalt (II) chloride (CoCl_2_.6H_2_O) | 23.8 µgL^-1^ | L-phenylalanine | 40 mgL^-1^ |
| Sodium molybdate dehydrate (NaMoO_4_.2H_2_O) | 24.2 µgL^-1^ | L-proline | 300 mgL^-1^ |
| Potassium iodate (KIO_3_) | 10.8 µgL^-1^ | L-serine | 60 mgL^-1^ |
| Myo-Inositol | 100 mgL^-1^ | L-threonine | 75 mgL^-1^ |
| Pyridoxine hydrochloride | 2 mgL^-1^ | L-tryptophan | 10 mgL^-1^ |
| Nicotinic acid | 2 mgL^-1^ | L-tyrosine | 10 mgL^-1^ |
| Ca-panthothenate | 1 mgL^-1^ | L-valine | 30 mgL^-1^ |
| Thiamine hydrochloride | 0.5 mgL^-1^ | Ergosterol | 15 mgL^-1^ |
| p-amino benzoic acid | 1 mgL^-1^ | Tween 80 | 0.5 mLL^-1^ |
| Riboflavin | 1.2 mgL^-1^ | Glutathione | mgL^-1^ |
| Biotin | 0.125 mgL^-1^ | Diammonium phosphate | 352 mgL^-1^ |
| Folic acid | 0.2 mgL^-1^ |  |  |
