# Supplementary figures and images for: Sporulation in soil as an overwinter survival strategy in Saccharomyces cerevisiae
Source: FEMS Yeast Res. 2015 Nov 13;16(1):fov102. doi: 10.1093/femsyr/fov102 (PMC5815064; doi:10.1093/femsyr/fov102)

## Agar

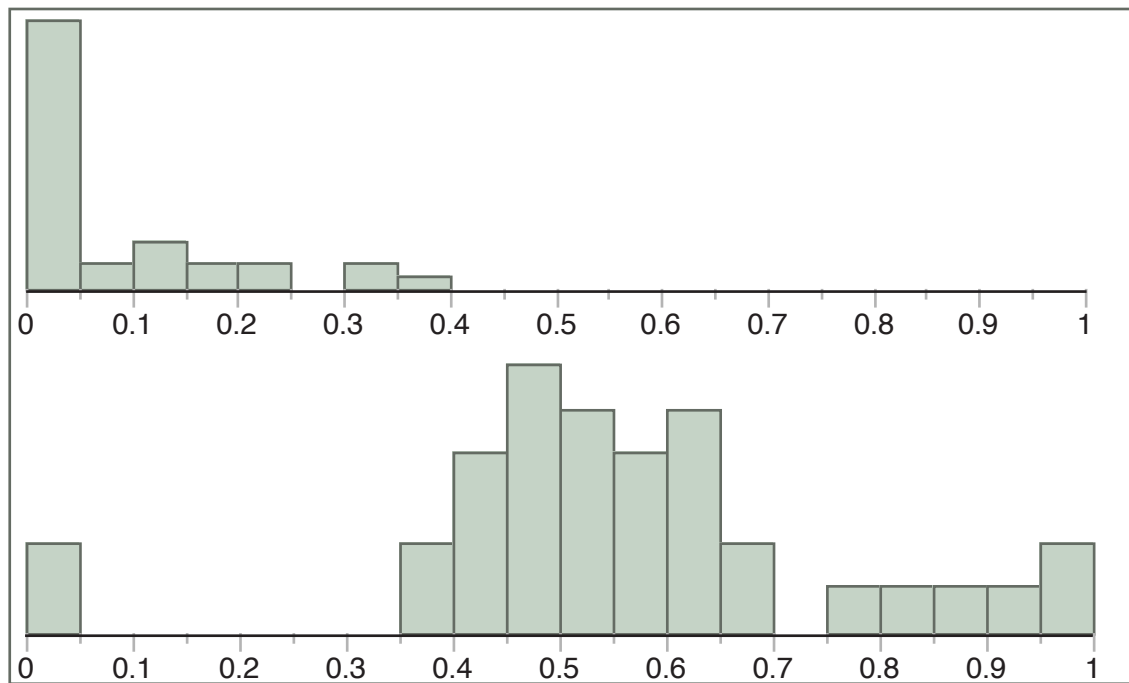

## Soil

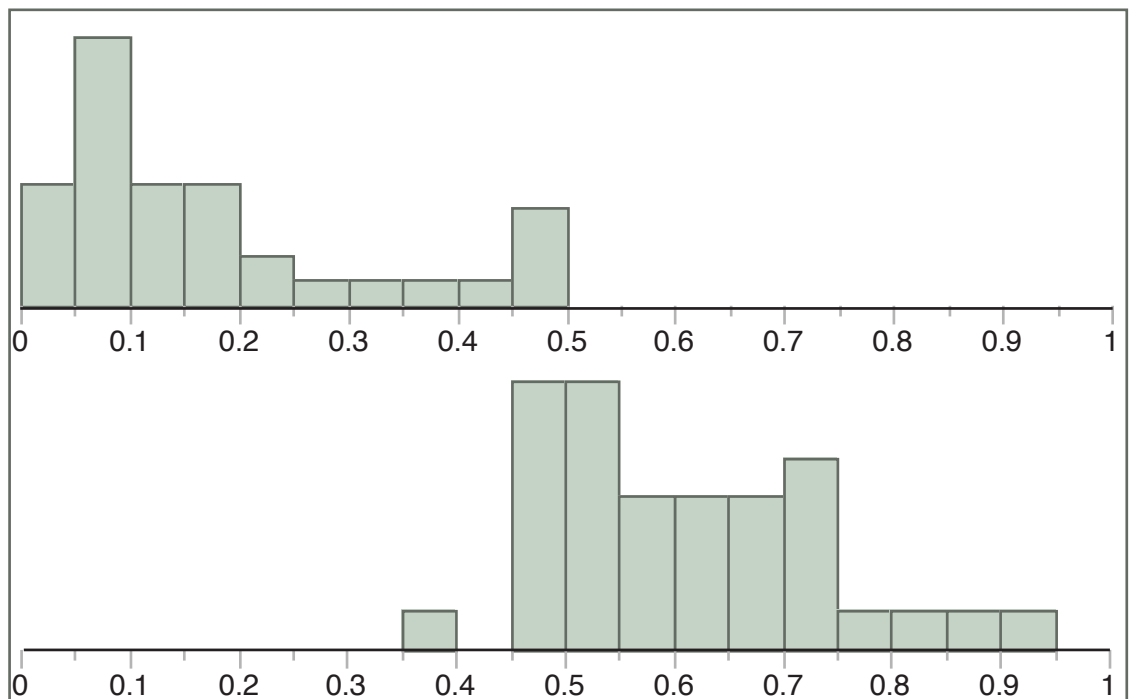

## Sporulation media

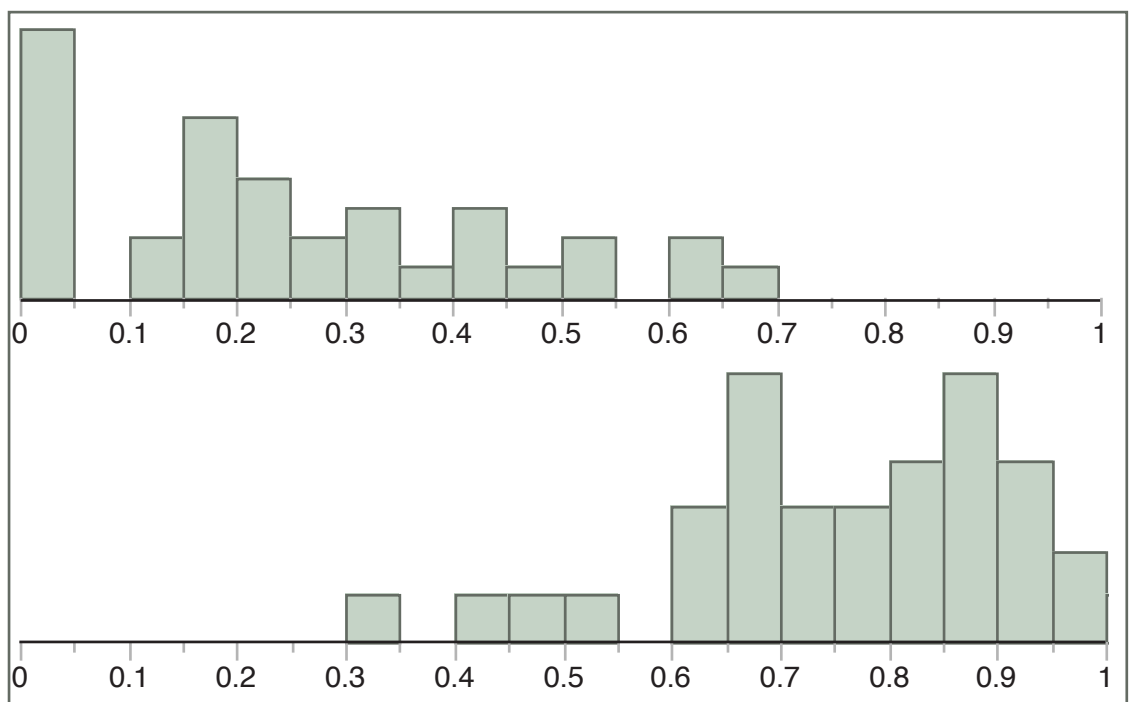

Supplement: Supplementary Data [file fov102_supplementary_data.zip › Supplementary Figure 1.pdf]

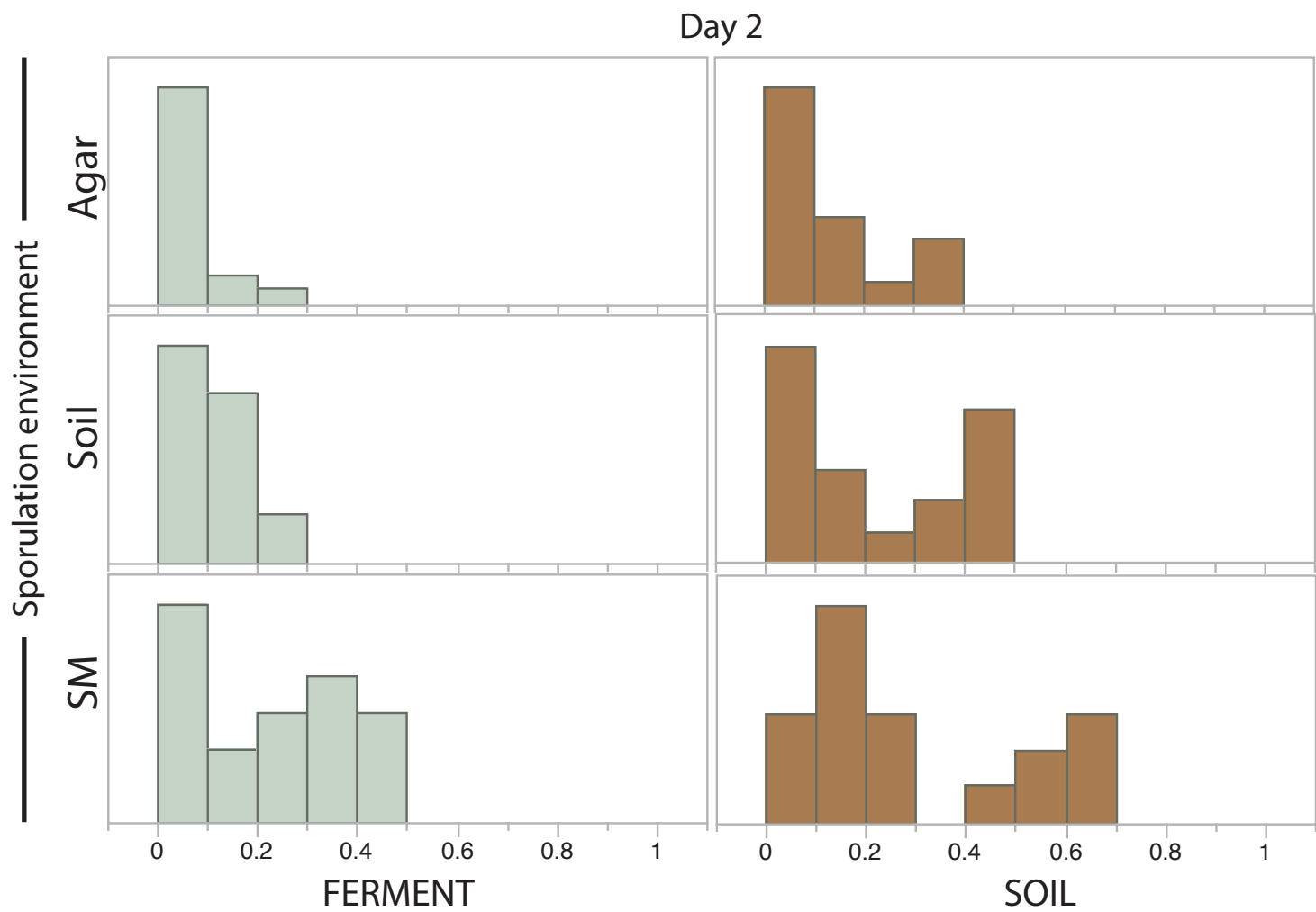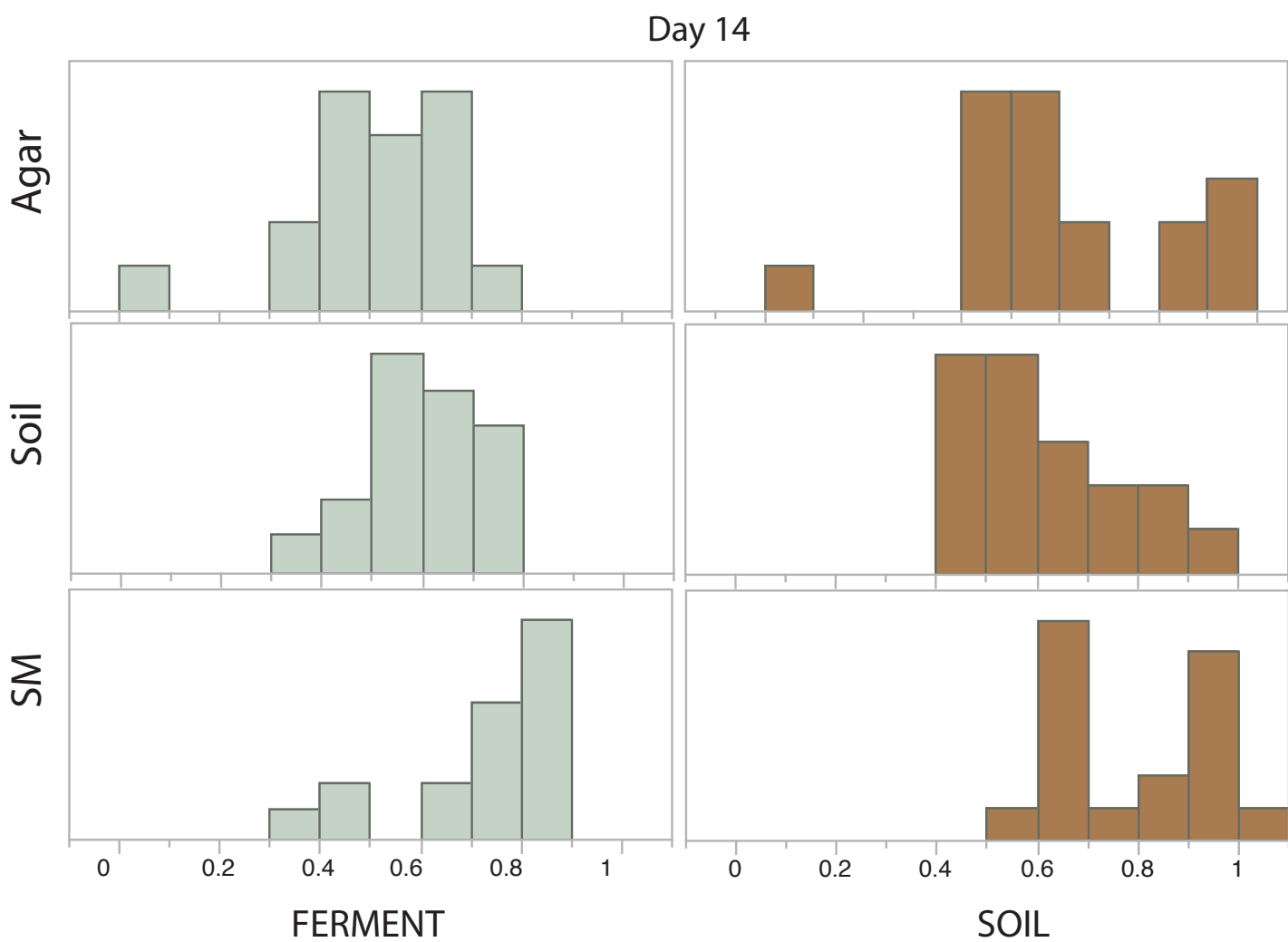

Isolate origin

Supplement: Supplementary Data [file fov102_supplementary_data.zip › Supplementary Figure 2.pdf]
